# The relationship between personality and success in language production tasks in pre-medical education

**DOI:** 10.3389/fmed.2026.1847054

**Published:** 2026-06-24

**Authors:** Abdulrahman Almalki, Mohammed Alzahrani

**Affiliations:** 1 English Language Department, College of Science and Health Professions, King Saud bin Abdulaziz University for Health Sciences, Riyadh, Saudi Arabia; 2 King Abdullah International Medical Research Center, Riyadh, Saudi Arabia

**Keywords:** academic writing, Big Five personality traits, communication, medical students, oral skills

## Abstract

Effective communication is a foundational competency for healthcare professionals, directly impacting clinical safety and interprofessional collaboration. This study investigated the impact of the Big Five personality traits (openness to experience, conscientiousness, extraversion, agreeableness, and neuroticism) on the communication skills of future medical practitioners. A cohort of 139 male pre-medical/health sciences students completed the 44-item Big Five Inventory (BFI-44) alongside academic writing and oral presentation tasks. Spearman’s rho correlation analyses revealed that openness to experience and extraversion were associated with success in both writing and speaking. Openness demonstrated significant positive correlations with writing (*r* = 0.292, *p* < 0.001) and oral skills (*r* = 0.310, *p* < 0.001), while extraversion also correlated significantly with both modalities. In contrast, conscientiousness, agreeableness, and neuroticism did not significantly correlate with performance in these specific communicative tasks. Additionally, a strong positive correlation (*r* = 0.500, *p* < 0.001) was identified between writing and oral proficiency, which suggests that these competencies may share underlying cognitive processes. These results highlight the critical role of non-cognitive factors in medical education and indicate that identifying stable personality profiles can help educators provide targeted support for the diverse communicative needs of pre-medical students.

## 1 Background

Effective communication is a foundation of clinical practice and a necessary competency for medical professionals. In healthcare, the ability to articulate complex scientific information, document patient histories accurately, and present clinical findings to other professionals or patients is essential for ensuring patient safety and promoting professional collaboration. While cognitive ability and general intelligence have traditionally been viewed as the primary determinants of academic performance (1, 2), recent research emphasizes the predictive power of non-cognitive factors, particularly personality, in determining professional success (3, 4). The Big Five personality traits, openness to experience, conscientiousness, extraversion, agreeableness, and neuroticism (OCEAN), serve as stable psychological markers that can predict how students approach learning and practice professional communication. Because these personality traits are stable over time, understanding their interplay with language production allows educators to identify students who may require targeted support to meet the rigorous communicative demands of the medical field (5).

In the context of medical education, generational shifts in medical education have prompted a move toward evaluating undergraduates’ soft skills from the start of their training, with research indicating that students feel a strong need to understand their own personal growth alongside academic study (6). These soft skills are increasingly assessed through the Big Five personality traits, which serve as stable psychological markers that can predict how students approach and practice professional communication. Within this framework, research suggests that agreeableness, openness, and extraversion are linked to superior communication skills. For example, Franco et al. (7) found that among Brazilian medical students, agreeableness demonstrated the strongest positive association with attitudes toward learning communication skills. This finding is further reinforced by a pilot study of Romanian medical students, where agreeableness correlated with favorable attitudes toward patient communication and showed a robust association with empathy (8). Among practicing physicians, this trait continues to correlate with better communication practice (9) and fosters greater trust and treatment adherence in patients (10, 11). Openness to experience also serves as a significant predictor of communicative success, as studies have shown that students high in openness possess the creativity and flexibility necessary for high-level language production, such as academic writing and oral presentations (12, 13). In medical settings, openness independently predicts positive attitudes toward communication training (7) and is a key driver of patient-centered communication (14). Furthermore, openness is positively correlated with empathy, which is an important mediator of effective patient-physician interactions (15). Similarly, extraversion is closely linked to a student’s willingness to communicate (WTC), a central factor for developing professional oral proficiency (12, 16). Additionally, extroverted individuals are naturally inclined to engage in the social-communicative acts (17) required for clinical practice.

On the contrary, conscientiousness plays a more nuanced role in medical education. While it is often a strong predictor of overall academic GPA (18), its impact on communication is complex. High conscientiousness can lead to thoroughness in exploring a patient’s psychosocial context, but it may also result in less collaborative treatment discussions (8, 14). Furthermore, while conscientiousness facilitates discipline in language practice (19), it may have a neutral or even negative relationship with spontaneous or creative tasks (20). Finally, neuroticism is consistently associated with negative outcomes, such as poorer performance in clinical objective structured clinical examination dimensions (21) and a reduced likelihood of involving patients in shared decision-making (9, 14).

Despite these global trends, there are inconsistencies across cultural and linguistic contexts (22). In Saudi Arabia, where English is the lingua franca for medical training, students must master complex language production tasks to succeed professionally. For these students, academic writing and oral presentations are not isolated skills; they share underlying cognitive processes and are mutually reinforcing (23, 24). Moreover, a gap remains in the literature regarding the comparison of personality traits across written and oral modes within the context of Saudi medical education. Much of the existing literature focuses on general academic achievement or on specific traits like WTC. The current study aims to address this lack of task-specific and regional data by investigating the relationship between the Big Five personality traits and the professional communication performance of pre-med students. This research seeks to provide an understanding that may assist educators in tailoring instructional approaches to support the diverse communicative needs of future healthcare professionals. This study is guided by the following research questions:

Research Question 1: To what extent do the Big Five personality traits correlate with the academic performance of pre-med/health sciences students in two language production modes (academic writing and oral presentation)?
Research Question 2: What is the relationship between students’ proficiency in academic writing and their oral presentation performance?

## 2 Methods

### 2.1 Participants

The current study included 139 first-year Saudi male college students enrolled in a university pre-med/health sciences program. The participants were randomly selected for the study. Participants were low-intermediate to intermediate English language learners, measured by the university’s English Placement Test (EPT). Students’ participation was voluntary, and they were notified of their freedom to withdraw during the data collection process. Students’ names and identifying information were replaced with pseudonyms and pseudo-identifying information. Students were not compensated for participating in this study. The study was approved by the institutional review board at King Abdullah International Medical Research Center in King Saud bin Abdulaziz University for Health Sciences, Riyadh, Saudi Arabia, protocol number NRR26/001/3.

### 2.2 Instruments

This study includes the Big Five Inventory questionnaire to measure personality traits. Specifically, this investigation utilized the 44-item personality trait questionnaire to assess the participants’ scores in the five dimensions (OCEAN) (25). The full questionnaire is provided in Appendix A. The participants’ L2 writing was measured through their final in-class writing task, where they were presented with a list of topics from which they chose one and were asked to write an expository piece. Their performances were evaluated based on the following categories: organization, content, and mechanics (Appendix B). Because the evaluation of writing is a subjective process, each paper was evaluated and validated by two language instructors, and any discrepancies and disagreements were resolved. In relation to oral skills measures, participants conducted an in-class presentation during Week 10 of the semester on a topic of their choice. They were assessed based on two main categories: the content and delivery of the presentation. Furthermore, these categories included the participant’s ability to organize the content logically and coherently, support their presentation with examples, use of sources and references, pronunciation, appropriateness of register (formality), and time management (Appendix C).

### 2.3 Procedure

This study was conducted over a full academic semester. The participants were randomly selected and recruited. Before data collection began, participants were notified that their involvement in the study was voluntary and they maintained the right to withdraw at any time. In Week 1, participants were administered the 44-item Big Five Inventory (BFI-44) via Google Forms. Throughout the semester, students engaged in intensive English coursework focused on developing high-level academic writing and oral communication skills. In Week 10, participants delivered an in-class oral presentation on a topic of their choice. Evaluations were conducted by two language instructors using a standardized rubric assessing content and delivery. To minimize bias, instructors were blinded to the students’ personality scores during the evaluation process. In week 12, participants completed a final in-class writing task. Participants were to choose a topic from a provided list of topics and produce an expository piece within 3 h. The scores in both academic writing and presentation were collected and analyzed in correlation with their personality trait scores.

### 2.4 Analysis

To establish a baseline profile of the participants, descriptive statistics [means, (*M*), and standard deviations, (*SD*)] were calculated for all seven variables: The five personality traits (OCEAN) and the two language production scores (academic writing and oral presentation). This allowed for the assessment of the overall personality trends and proficiency levels of the two selected areas within the sample. Personality scores were calculated using a 5-point Likert scale. Sixteen specific items (2, 6, 8, 9, 12, 18, 21, 23, 24, 27, 31, 34, 35, 37, 41, and 43) were reverse-scored to ensure all responses aligned with the respective trait dimensions. For the final analysis, scores for each dimension were calculated as item-level means (1–5 scale). Table 1 presents the descriptive statistics for the participants’ scores across the five personality dimensions, writing skills, and oral skills. The participants were assessed for their personality traits with mean scores for each trait as follows: Openness to experience (*M* = 3.42, *SD* = 0.48), conscientiousness (*M* = 3.60, *SD* = 0.65), extraversion (*M* = 3.10, *SD* = 0.61), agreeableness (*M* = 3.82, *SD* = 0.52), and neuroticism (*M* = 2.78, *SD* = 0.73). In addition, the participants’ performance in writing and oral skills was evaluated, which yielded mean scores of 91.93 (*SD* = 6.34) for writing and 94.29 (*SD* = 7.06) for oral skills. These results indicate generally high levels of both writing and oral proficiency among participants, with the average oral score slightly higher than the average writing score.

**TABLE 1:** Descriptive statistics of scores across the five personality traits, writing, and oral skills.

Personality trait	*N*	*M*	*SD*
Openness to experience	139	3.42	0.48
Conscientiousness	139	3.60	0.65
Extraversion	139	3.10	0.61
Agreeableness	139	3.82	0.52
Neuroticism	139	2.78	0.73
Writing	139	91.93	6.34
Oral skills	139	94.29	7.06

Personality trait scores are reported as item-level means (scale 1–5), and writing and oral presentation scores are reported as percentages (0–100).

To address the first research question in relation to the relationship between personality traits and language production skills, Spearman’s rho correlation analyses were performed. Because the data for several variables were not normally distributed, non-parametric Spearman’s rho correlation analyses were employed instead of Pearson correlations to ensure statistical validity. This statistical test was used to identify the strengths and direction of the relationship between each of the Big Five traits and the participants’ scores in academic writing and oral presentation. Significance for these correlations was measured at both the 0.05 and 0.01 levels to determine which personality traits significantly correlate with academic performance. In relation to the second research question, a correlation analysis was conducted to explore the relationship between EFL learners’ writing proficiency and their oral presentation performance. This analysis aimed to determine whether these two distinct modes of language production are significantly correlated.

## 3 Results

Table 2 presents Spearman’s rho correlation coefficients for the relationships between the Big Five personality traits and students’ academic writing and oral skills scores. This table highlights the significant positive correlations of openness to experience and extraversion in relation to both writing and oral performances. On the other hand, conscientiousness, agreeableness, and neuroticism were not shown to have significant correlations with the scores. The correlation coefficient is presented first and followed by the significance in the subsequent row.

**TABLE 2:** Spearman’s rho correlations of writing and oral scores and the Big Five personality traits.

Personality trait	O	C	E	A	*N*	Writing	Oral
Openness to experience	1.000	0.116	0.136	0.179*	−0.078	0.292**	0.310**
	–	0.174	0.111	0.035	0.363	>0.001	>0.001
Conscientiousness	0.116	1.000	0.233**	0.301**	−0.328**	−0.069	−0.058
	0.174	–	0.006	>0.001	>0.001	0.418	0.501
Extraversion	0.136	0.233**	1.000	−0.014	−0.152	0.171*	0.205*
	0.111	0.006	–	0.868	0.075	0.044	0.015
Agreeableness	0.179*	0.301**	−0.014	1.000	−0.252**	0.031	−0.031
	0.035	>0.001	0.868	–	0.003	0.717	0.718
Neuroticism	−0.078	−0.328**	−0.152	−0.252**	1.000	−0.033	−0.080
	0.363	>0.001	0.075	0.003	–	0.702	0.348

*Correlation is significant at the 0.05 level (2-tailed).

**Correlation is significant at the 0.01 level (2-tailed).

The analysis of the relationship between the Big Five personality traits and students’ writing and oral presentation scores revealed several significant correlations. Openness to experience was positively correlated with both writing (*r* = 0.292, *p* < 0.01) and oral scores (*r* = 0.310, *p* < 0.01). This indicates that students with higher levels of Openness tend to perform better in both writing and presentation tasks. Similarly, extraversion showed significant positive correlations with writing (*r* = 0.171, *p* < 0.05) and oral scores (*r* = 0.205, *p* < 0.05). This suggests that students with higher extraversion tend to achieve higher scores in both skills. In contrast, conscientiousness, agreeableness, and neuroticism did not demonstrate significant relationships with writing and oral presentation scores. Specifically, conscientiousness had weak negative correlations with both scores (writing: *p* = 0.418; oral: *p* = 0.501), while agreeableness (writing: *p* = 0.717; oral: *p* = 0.718) and neuroticism (writing: *p* = 0.702; oral: *p* = 0.348) showed no meaningful correlations with either skill. Regarding the relationship between writing performance and oral presentation, the findings presented in Table 3 suggest a significant correlation (*r* = 0.500, *p* < 0.01). This indicates that higher performance in academic writing is associated with better oral presentation skills.

**TABLE 3:** Spearman’s rho correlations of writing and oral scores.

Skill	Writing	Oral
Writing	1.000	0.500**
	–	>0.001
Oral skills	0.500**	1.000
	>0.001	–

**Correlation is significant at the 0.01 level (2-tailed).

The interrelationships within the personality traits (Table 2) showed that conscientiousness was positively correlated with extraversion (*r* = 0.233, *p* < 0.01) and agreeableness (*r* = 0.301, *p* < 0.01). Conversely, conscientiousness had a significant negative correlation with neuroticism (*r* = −0.328, *p* < 0.01). Openness had a small but significant positive correlation with agreeableness (*r* = 0.179, *p* < 0.05), and extraversion displayed a negligible relationship with agreeableness (*r* = −0.014, *p* = 0.868) and a weak negative correlation with Neuroticism (*r* = −0.152, *p* = 0.075). Lastly, neuroticism showed significant negative correlations with both agreeableness (*r* = −0.252, *p* < 0.01) and conscientiousness.

## 4 Discussion

This study investigated the impact of the Big Five personality traits on the professional communication skills of Saudi pre-med/pre-health sciences students. The results revealed that openness to experience and extraversion are the primary personality dimensions that are associated with success in these high-stakes language production modes. The findings can provide a framework for educators to identify students who may require targeted support to meet the communicative demands of the medical profession (5). Specifically, openness to experience demonstrated a significant positive correlation with both writing (*r* = 0.292) and oral skills (*r* = 0.310). This aligns with prior evidence that suggests individuals high in openness possess the creativity and flexibility necessary for proficient writing and complex cognitive tasks (12, 13). In a medical context, this trait is recognized as a key driver of patient-centered communication (14) and is positively correlated with empathy, which is a vital mediator of effective patient-physician interactions (15). Similarly, extraversion was positively correlated with both modes of production. This supports the notion that extroverted learners are more likely to invest effort in language acquisition and engage in the social-communicative tasks required for clinical practice (17). Extraversion is closely linked to a student’s WTC, which is an important factor for developing professional oral proficiency (12, 16). While these medical students can be characterized as ambitious problem-solvers due to high scores in extraversion and openness, they may score lower on relational traits, highlighting the importance of these findings for communication training (7, 26).

Interestingly, conscientiousness, agreeableness, and neuroticism showed no significant correlations with performance in this study. The lack of significance of conscientiousness for participants’ performance in both written and spoken language production tasks runs counter to prior literature [e.g., (6)]. While it is often associated with overall academic success (18), traditional markers of intelligence and conscientiousness may not always translate directly to communicative competence (1). The nature of the tasks in this study, specifically the writing task, requires spontaneous language production, as students were informed of the topics they could choose during data collection. The time-limited in-class nature of the assessment may have favored flexibility over the long-term discipline typically associated with high conscientiousness. While conscientiousness is typically the strongest predictor of overall academic GPA, it may be less relevant for isolated, task-specific performance. This may explain the findings of the current study, as conscientiousness can have a neutral or even negative relationship with tasks that favor spontaneity over structured rote learning (20).

Furthermore, while agreeableness is consistently linked to positive attitudes toward learning communication skills (7), empathy (8), and better clinical practice among physicians (9), it did not significantly correlate with language production scores in this cohort. While agreeableness may have a more favorable disposition toward patient interaction (10, 11), this interpersonal orientation may not translate to higher technical proficiency in academic writing or formal oral presentations.

Finally, neuroticism, which is typically associated with poorer objective structured clinical examination performance (27) and a reduced tendency to involve patients in shared decision-making (14), did not significantly correlate with the task scores. This may be due to the specific testing environment; the in-class nature of these assessments likely did not reach the high stress of clinical exams, thereby neutralizing the negative performance interference that may be triggered by high neuroticism.

An important finding of this research is the positive correlation between writing and oral presentation scores. This result confirms that academic writing and speaking skills may share underlying cognitive processes and are mutually reinforcing in a professional medical curriculum (23, 24). Students who exhibit proficiency in the logical organization and mechanics of a written report are statistically likely to demonstrate similar competence in oral delivery. This interconnectedness highlights the necessity of an integrated approach for language learners and professional communication training for future healthcare providers.

## 5 Conclusion

This study investigated the role personality traits play in relation to language production. The results demonstrated that openness to experience and extraversion significantly correlated with higher scores in both academic writing and oral presentation tasks. Students with higher scores in these two personality traits tended to perform better in these language production tasks (academic writing task and oral presentation). In contrast, traits such as conscientiousness, agreeableness, and neuroticism did not show a significant relationship with performance in this specific context. Furthermore, the study established a strong positive correlation between writing and speaking proficiency; proficiency in one can correlate with performance in the other.

The findings of the current study offer insights for EFL pedagogy and curriculum design. Educators could cultivate learning environments that foster curiosity and social interaction to help students leverage traits such as openness and extraversion. Medical educators should move beyond traditional cognitive measures to consider individual personality profiles when designing support systems and curricula for future healthcare professionals. Additionally, the link between writing and oral performance may suggest that language programs could adopt an integrated approach to teaching both of these skills. Understanding such individual differences among students could also allow educators to tailor their approach to support students with diverse communicative needs.

One of the limitations of this study is that the sample included 139 male students from a pre-health scientific majors program, which may limit the generalizability of the results to female students or learners in different programs. Future studies could include female students as well as students from diverse academic fields. The study also used Spearman’s rho correlation analysis, which identifies the strength and direction of relationships between personality traits and language scores. However, this method establishes associations but not causality and does not determine the relative predictive weight of each trait when considered simultaneously with others. Moreover, the statistical relationships identified may be specific to the nature of the tasks used in this study. Personality traits might play different roles in other types of language-production tasks. Future studies could utilize different language production tasks to show how personality traits interplay with diverse language production tasks. Finally, the relationship between personality traits and performance may be dependent on the task. For instance, consciousness may play a more important role in a different language-production task as opposed to the ones used in this semester-long study.

## Data Availability

The raw data supporting the conclusions of this article will be made available by the authors, without undue reservation.

## Appendix A | The Big Five Inventory (BFI-44).

**Table:**

Here are a number of characteristics that may or may not apply to you. For example, do you agree that you are someone who likes to spend time with others? Please write a number next to each statement to indicate the extent to which you agree or disagree with that statement.					
1. Disagree strongly			_	20	Has an active imagination
2. Disagree a little			_	21	Tends to be quiet
3. Neither agree nor disagree			_	22	Is generally trusting
4. Agree a little			_	23	Tends to be lazy
5. Agree strongly			_	24	Is emotionally stable, not easily upset
*I see myself as someone who…*			_	25	Is inventive
_	1	Is talkative	_	26	Has an assertive personality
_	2	Tends to find fault with others	_	27	Can be cold and aloof
_	3	Does a thorough job	_	28	Perseveres until the task is finished
_	4	Is depressed, blue	_	29	Can be moody
_	5	Is original, comes up with new ideas	_	30	Values artistic, aesthetic experiences
_	6	Is reserved	_	31	Is sometimes shy, inhibited
_	7	Is helpful and unselfish with others	_	32	Is considerate and kind to almost everyone
_	8	Can be somewhat careless	_	33	Does things efficiently
_	9	Is relaxed, handles stress well	_	34	Remains calm in tense situations
_	10	Is curious about many different things	_	35	Prefers work that is routine
_	11	Is full of energy	_	36	Is outgoing, sociable
_	12	Starts quarrels with others	_	37	Is sometimes rude to others
_	13	Is a reliable worker	_	38	Makes plans and follows through with them
_	14	Can be tense	_	39	Gets nervous easily
_	15	Is ingenious, a deep thinker	_	40	Likes to reflect, play with ideas
_	16	Generates a lot of enthusiasm	_	41	Has few artistic interests
_	17	Has a forgiving nature	_	42	Likes to cooperate with others
_	18	Tends to be disorganized	_	43	Is easily distracted
_	19	Worries a lot	_	44	Is sophisticated in art, music, or literature

BFI scale scoring (“R” denotes reverse-scoring items): Extraversion: 1, 6R, 11, 16, 21R, 26, 31R, 36; Agreeableness: 2R, 7, 12R, 17, 22, 27R, 32, 37R, 42; Conscientiousness: 3, 8R, 13, 18R, 23R, 28, 33, 38, 43R; Neuroticism: 4, 9R, 14, 19, 24R, 29, 34R, 39; Openness: 5, 10, 15, 20, 25, 30, 35R, 40, 41R, 44.

## Appendix B | Academic Writing Assessment Rubric.

**Table:**

Category	Evaluation criteria	Score
Organization	Topic Sentence: Clear and strong; includes both topic and controlling idea.	
	Support & Detail: At least three supporting sentences for main points and three detail sentences for explanation.	
	Conclusion: Clear and strong concluding sentence.	
Content	Task requirements: On topic and meets specific rhetorical requirements.	
	Unity: Discusses only one topic; all sentences are directly related.	
	Coherence: Logical order; consistent use of nouns/pronouns; correct transition signals.	
	Vocabulary range: Uses higher-level vocabulary appropriate to the topic.	
	Sophistication: Avoids informal language/repetition; demonstrates deep idea development.	
Mechanics	Punctuation: Proper use of end marks, commas, and capital letters.	
	Verbs: Correct subject/verb agreement, tenses, and auxiliary verb usage.	
	Lexical accuracy: Correct word choice, parts of speech, and singular/plural forms (including articles/prepositions).	
	Spelling: All words are spelled correctly.	
	Sentence structure: Variety of simple and compound sentences; no fragments; correct word order.	
	Grammatical accuracy: Free of any other general grammatical errors.	
Total Score		

## Appendix C | Oral Presentation Assessment Rubric.

**Table:**

	Criteria	Great	Average	Below average	Poor
Content	Organization	Seamless, highly logical flow	Logical and coherent overall	Some structure; lacks cohesion	No logical flow; confusing
	Introduction	Engaging and clear hook/thesis	Clearly stated from the start	Topic stated but unclear	Topic/ideas not stated
	Body (Evidence)	Rich, highly relevant examples	Sufficient details and examples	Few examples provided	Lacks details or examples
	Conclusion	Memorable and concise summary	Planned and fitting conclusion	Vague closing remarks	Abrupt or missing
	Referencing	3 + high-quality sources used	Minimum 2 sources provided	1 source provided	0 sources provided
Delivery	Vocal Fluency	Natural flow; expert intonation	Minimal fillers; appropriate pauses	Noticeable fillers; inconsistent pauses	Frequent fillers (uhm); poor pauses
	Volume	Clear and effective projection	Loud enough to be heard	Inconsistent volume	Too quiet to be heard
	Pronunciation	Perfect pronunciation and clarity	Mostly correct and clear	Several errors; occasionally unclear	Many errors; hard to understand
	Language Use	Sophisticated and accurate	Generally correct grammar/vocab	Noticeable L2 errors	Many grammar/vocab errors
	Style & Contact	Confident; maintained eye contact	Looked at slides occasionally	Constant reliance on notes	Read entirely from slides
	Visual Aids	Professional and effective	Easy to read; balanced visuals	Inconsistent quality	Overloaded/blank; unreadable
	Time Management	Perfect timing and pacing	Within 3–5 min (½ min window)	Slightly over/under time	Significant deviation from time
